# Moving Scabies Under Video-Dermoscopy

**DOI:** 10.4269/ajtmh.25-0451

**Published:** 2025-12-16

**Authors:** Meirong Li, Huaiqiu Huang, Peiying Feng

**Affiliations:** Department of Dermatology and Plastic surgery, Third Affiliated Hospital, Sun Yat-Sen University, Guangzhou, China

Norwegian (crusted) scabies is a highly contagious form of scabies in which hyperkeratotic, scaly, highly pruritic lesions teem with mites. This syndrome usually occurs in immune-compromised individuals or in elderly, cognitively impaired, or bedridden individuals.^1^ Because the condition typically progresses from ordinary scabies, most patients still exhibit classic scabies rashes, such as scattered papulo-vesicles and burrows. The diagnosis is typically established by direct examination of mites or ova from scapings and/or biopsy visualized under light microscopy. With technological advancements, diagnostic methods such as Wood’s lamp examination and dermoscopy have been successively introduced, significantly improving the accuracy of clinical diagnoses.^2^^,^^3^ Dermoscopy and video-dermoscopy have the potential to enhance efficient and accurate diagnosis of scabies.

A 76-year-old wheelchair-bound man was admitted to the hospital with a 6-month history of generalized eczematous and pruritus dermatitis. Rash was observed on the neck, trunk, scrotum, buttocks, and extremities. Both wrists exhibited hyperkeratotic, fissured, oyster-shell–like plaques devoid of vesicles.

Two modalities were used to observe the rashes. Wood’s lamp (UV light) examination was used to observe the fluorescent reaction of the mites and their burrows (Figure 1A). Dermoscopy was performed under polarized light (20×, Dermat, Beijing, China); and the images were captured with a smartphone. Polarized dermoscopy showed the sinuous burrow and the brown jet-shaped triangular structure of a translucent mite at the ends of the burrow (Figure 1E). Mites were observed moving rapidly and irregularly (recorded under video-dermoscopy) (Supplemental Video 1). With the guidance of dermoscopic and video-dermoscopic findings, direct microscopic examination confirmed the diagnosis of crusted scabies by the direct observation of mites and their burrows (Figure 1C). The morphology of the mites, their sinuous burrows, and their dynamic movements was notable.^4^^,^^5^

**Figure 1.**
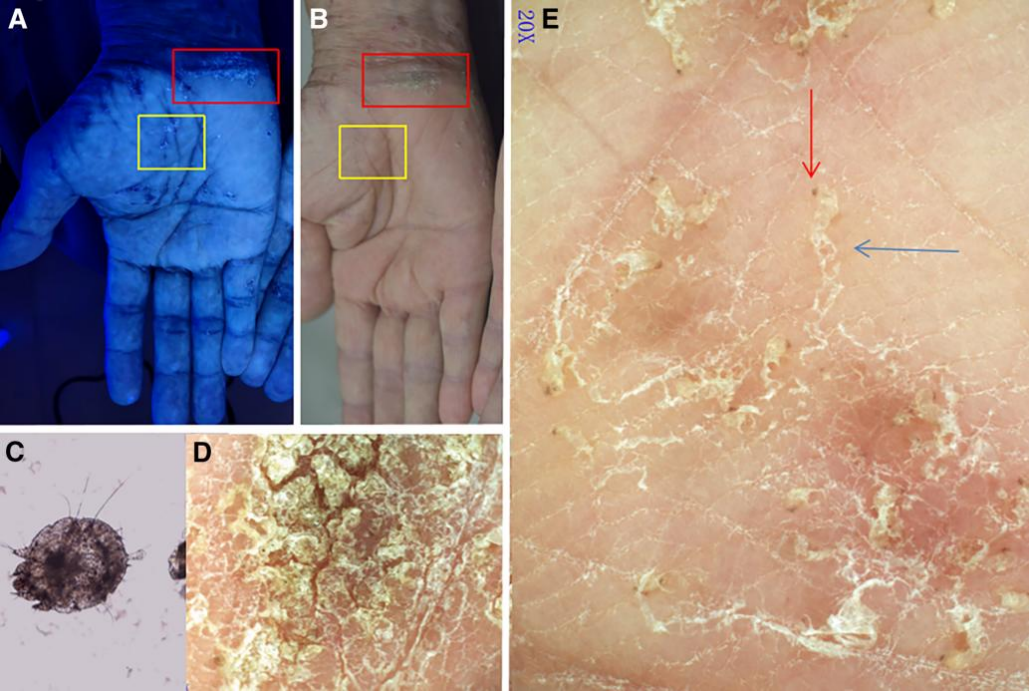
(**A**) Under Wood’s lamp examination, extensive blue-white linear fluorescent lesions are observed on the palms, while blue-bright white crusted lesions are noted on the wrists. (**B**) Grayish-white burrows are visible on the palms and between the fingers, while crusted lesions are observed on the wrists. (**C**) Microscopic direct examination reveals the presence of Sarcoptes scabiei mites (original magnification ×40). (**D**) The lesion within the red box is magnified. Hands with thick crusts and deep fissures on the wrist (original magnification ×20). (**E**) The lesion within the yellow box is magnified. Polarized dermoscopic images of left palm lesion showed multiple sinuous burrow (blue arrow) and brownish triangular structure at the end of the burrow (red arrow).

The movements of sarcoptic mites on the surface of the skin observed by dermoscopy are different than the static state observed by microscopy. This visualization of actively moving mites rapidly confirms its highly contagious nature and enables efficient initiation of infection control practices.

This patient was successfully treated with a continuous three-day application of 10% sulfur ointment without bathing. An alternative treatment could have been topical permethrin or oral ivermectin. No other cause of immunocompromise was identified. To eliminate the risk of interpersonal transmission, close contacts were required to wear disposable gloves during any physical interaction, and where appropriate, received prophylactic therapy.

## Supplemental Materials

10.4269/ajtmh.25-0451 Supplemental Materials
